# Reducing risk in the emergency department: a 12‐month prospective longitudinal study of radiographer preliminary image evaluations

**DOI:** 10.1002/jmrs.341

**Published:** 2019-08-26

**Authors:** Cameron Brown, Michael J. Neep, Efrosini Pozzias, Steven M. McPhail

**Affiliations:** ^1^ Department of Medical Imaging Logan Hospital Corner of Armstrong Road and Loganlea Road Meadowbrook Queensland Australia; ^2^ School of Public Health and Social Work Queensland University of Technology Kelvin Grove Brisbane Australia; ^3^ Institute of Health and Biomedical Innovation Queensland University of Technology Kelvin Grove Brisbane Australia; ^4^ Centre for Functioning and Health Research Metro South Health Buranda Plaza, Corner Ipswich Road and Cornwall Street Buranda Brisbane Australia

**Keywords:** General radiography, medical imaging, radiographer, skeletal/bones

## Abstract

**Introduction:**

Innovations are necessary to accommodate the increasing demands on emergency departments whilst maintaining a high level of patient care and safety. Radiographer Preliminary Image Evaluation (PIE) is one such innovation. The purpose of this study was to determine the accuracy of radiographer PIE in clinical practice within an emergency department over 12 months.

**Methods:**

A total of 6290 radiographic examinations were reviewed from 15 January 2016 to 15 January 2017. The range of adult and paediatric examinations incorporated in the review included the appendicular and axial skeleton including the chest and abdomen. Each examination was compared to the radiologist's report this allowed calculated mean sensitivity and specificity values to indicate if the radiographer's PIE was of a true negative/positive or false negative/positive value. Cases of no PIE participation or series’ marked as unsure for pathology by the radiographer were also recorded. This allowed mean sensitivity, specificity and diagnostic accuracy to be calculated.

**Results:**

The study reported a mean ± 95% confidence level (standard deviation) for sensitivity, specificity, accuracy, no participation and unsure of 71.1% ± 2.4% (6.1), 98.4% ± 0.04% (0.9), 92.0% ± 0.68% (1.9), 5.1% (1.6) and 3.6% (0.14) respectively.

**Conclusions:**

This study has demonstrated that the participating radiographers provided a consistent PIE service while maintaining a reasonably high diagnostic accuracy. This form of image interpretation can complement an emergency referrer's diagnosis when a radiologist's report is unavailable at the time of patient treatment. PIE promotes a reliable enhancement of the radiographer's role with the multi‐disciplinary team.

## Introduction

Within the emergency department (ED), medical imaging requests for plain radiography continue to rise, increasing the demand for a radiologist report.^1^ This growth in demand has frequently resulted in a suboptimal radiology reporting turnaround time.^2^ In particular, the emergency referrer is frequently required to make patient treatment decisions prior to the availability of the ‘gold standard’ radiology report. Existing evidence has raised concerns regarding the inexperience of some emergency referrers’ ability to interpret x‐rays, increasing the potential for inaccurate or incomplete patient management.^3^, ^4^, ^5^ This delay in the provision of a radiology report is becoming a progressive concern for patient safety. The formal reporting rate of emergency department radiographs at a tertiary hospital in Queensland, Australia was reviewed between 2011 and 2014. The report indicated that 238,453 radiographs had not received a definitive report by a radiologist.^6^ Furthermore, in this same report, in 2011–2012 a reporting rate as low as 42% was reported.^6^ Innovations to mitigate this risk to the patient are paramount to maintaining a high‐quality contemporary Australian health service.

There are numerous strategies that can be employed to reduce diagnostic errors and minimise treatment delays in the emergency department. One strategy that has been suggested by several authors is where a radiographer documents their interpretive opinion on the presence of potential pathology on the radiographs they acquire.^7^, ^8^, ^9^, ^10^ The timing of the radiographer's interpretation is particularly relevant when a radiologist report is not available in time to impact patient management.^11^ Where such a system is operational, the radiographer's interpretive opinion accompanies the images acquired, therefore is available immediately to the referrer. Radiographer interpretation systems have been implemented in the United Kingdom (UK) since 1985.^12^ The first system began by the radiographer marking any x‐ray film that was considered abnormal with a red sticker. This became known as the ‘Red Dot’ system.^12^ Although effective, the red dot system only indicates when an abnormality is present. It does not describe the abnormality, indicate when no interpretation is given, nor document if multiple abnormalities exist. This system evolved over the 1990s to incorporate a succinct comment of the radiographic findings by the practicing radiographer. This system became known in the UK as ‘Preliminary Clinical Evaluation’.^12^ This service, although limited in scope, has been suggested to reduce errors in diagnostic image interpretation within the emergency setting.^13^

A Preliminary Image Evaluation (PIE) has been described^14^ as an Australian system similar to the UK's Preliminary Clinical Evaluation, and does not replace the radiologist's report. Due to its timely availability, a PIE can assist an emergency referrer in their diagnosis within a clinically appropriate timeframe when the radiologist's report is delayed.^6^ Within Australia, a number of studies have investigated radiographer interpretation systems.^8^, ^10^, ^15^ These studies have involved a select group of radiographers with outcome measures conducted under laboratory (non‐clinical) conditions. Although these studies indicate promising benefits of a PIE system, clinical data and functional trials are pertinent to its development nationally and internationally. The purpose of this study was to determine the accuracy of radiographer PIE in clinical practice within an emergency department over 12 months.

## Methods

### Ethics

Metro South Hospital and Health Service Human Research Ethics Committee provided ethical approval. No personal identifying information was collected during this study. Participants were free to withdraw their consent at any time.

### Design

A prospective longitudinal study design was used to determine the accuracy of radiographer PIEs between 15 January 2016 to 15 January 2017 at Logan Hospital, Queensland, Australia.

### Study setting and participants

Logan hospital is located in southeast Queensland. The hospital has 448 beds within the Metro South Hospital Health Service.^16^ From July 2016 to June 2017, 88,256 patients presented to the hospital's emergency department, the second largest intake in the state.^17^, ^18^ Participants in this study included all radiographers who worked in Logan Hospital's emergency medical imaging department within the study period (*n* = 35). Radiographers were required to complete an in‐house image interpretation education programme within 10 weeks of commencing participation in the departmental PIE service. The programme required participants to complete 17 image interpretation modules that covered pathologies of the axial and appendicular skeleton, chest and abdomen within the PIE scope (see Table 1). These modules were reviewed by the x‐ray team leader before circulating to staff. Participants included radiography students whose interpretations were supervised by a qualified radiographer and were attributed to the registered professional. The PIE system was implemented 5 months prior to the commencement of this study. The PIE service was operational 24 hours a day, 7 days a week throughout the study period. This aspect of the study design was adopted to ensure the sample adequately represented radiographers’ case mix.

**Table 1:** Scope of preliminary image evaluation pathologies

The Radiographer's Scope A Bony Fracture A Joint Dislocation or Subluxation A Foreign Body A Pneumothorax A Pneumoperitoneum Knee Lipohaemarthrosis or Posterior Elbow Joint Effusion Any examinations which don't query any of these pathologies are outside scope and are not included in the audit.

### Audit sample size calculation

The research team took a pragmatic approach to determining an appropriate method to calculate the sample size of radiographic examinations to be assessed. The key objectives were to obtain a sample that was representative of a variety of radiographic examinations including different anatomical regions, performed at different times of the day by a variety of radiographers. It was decided to review all eligible radiographic examinations from 2 days every week; 1 weekday (Wednesday) and 1 weekend day (Sunday). This equated to approximately 120 examinations per week. At the time of the study, the total number of radiographic examinations performed in the emergency department in 2016 was 42,765. Consequently, the sampling method adopted in this study yielded approximately 14.7% of the annual examinations performed in the emergency department. Given a recommended sample size of 13.7% (90% Confidence level, ±1% confidence interval) was calculated using a published audit size calculator,^19^ this study's estimation of sample size was considered suitable.

### Procedure

**Figure 1:**
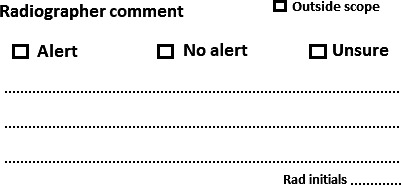
Preliminary image evaluation stamp.

The PIE system implemented into the ED at the study site involved a radiographer providing an immediate written opinion on the presence or absence of a potential traumatic or acute abnormality in relation to the radiographic examination they performed. The pathologies that were considered within the scope of the department's PIE system were set under the guidelines of the profession's national regulatory body^20^, ^21^ and are listed in Table 1. The referrer was required to specifically question the presence of one of the listed pathologies on the request form for it to be considered within scope. The radiographer was required to document their interpretation on the corresponding medical imaging request form via a novel stamp developed by the research team (Figure 1). Their interpretation of the pathology would constitute either an ‘alert’ (abnormality present), ‘no alert’ (no abnormality present), ‘unsure’ or ‘outside scope’. If the ‘alert’ box was ticked, a free text description was provided describing the abnormality that was detected. If ‘no alert’ was ticked, then it was assumed that the examination was considered normal and a free text description was not required. If the referrer's clinical question did not query any pathologies defined within the PIE's scope, then it was considered ‘outside scope’ and was marked accordingly.

**Figure 2:**
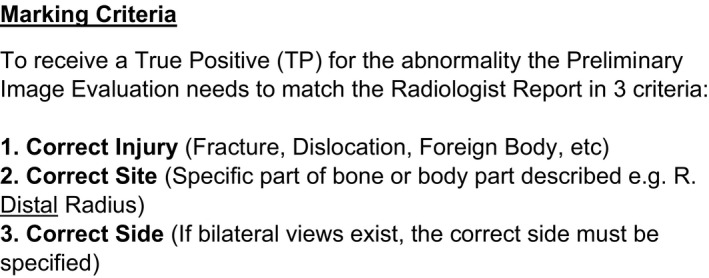
Marking criteria.

**Figure 3:**
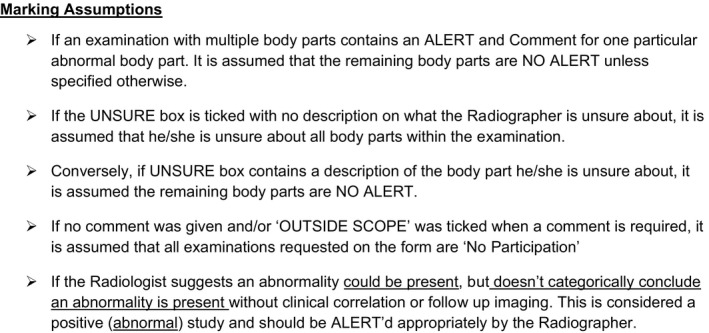
Marking assumptions.

Each week, a sample of the radiographers’ PIEs were reviewed by a single auditor for errors using a modified marking criteria (Figure 2) and list of marking assumptions that were developed by the research team (Figure 3). These were used to objectively assess the radiographer's PIE accuracy against the radiologist's report. It should be noted that the radiographer's PIE was visible to the radiologist at the time of reporting. From this comparison, each PIE was then allocated one of the following categories:

- True Positive (TP)
- True Negative (TN)
- False Positive (FP)
- False Negative (FN)
- Combined True Positive/False Negative (TP/FN)
- Unsure
- No PIE provided (non‐participation)

TP: The radiographer's PIE and the radiologist's report agreed on the presence of an acute abnormality, and on the description of the abnormality in the terms of injury, site and side. If multiple abnormalities were present, and one or more were missed by the radiographer, a TP score was not recorded. Similarly, credit was not given if the injury, site and side of the abnormality/ies was incorrectly described.

TN: The radiographer's PIE and the radiologist's report agreed on the absence of any acute abnormality. If a normal variant was identified by the radiographer and radiologist, a TN was allocated.

FP: The radiographer's PIE identified and described an appearance as abnormal, however, the radiologist's report disagreed and considered the examination to be normal, a FP was allocated.

FN: The radiographer's PIE identified an examination as normal, however, the radiologist's report stated that an acute abnormality was present.

TP/FN: If the radiographer identified the correct abnormality but did not provide all key elements of the description (type of injury, site and side) or multiple abnormalities that were included in the radiologist's report; however, not all were identified, then fractions of marks (1/2) were awarded. For any case, where partial marks were awarded, the constituent partial marks amount to one mark. For example if 1/2 TP was allocated, the remaining mark given was 1/2 FN.

Unsure: If the radiographer marked the unsure box within the PIE stamp, an unsure value was assigned (regardless if any interpretation was documented and whether it agreed or disagreed with the radiologist's report).

Non‐Participation: If the imaging request was within the radiographer's scope and required a PIE, but no interpretation was provided; a No Participation value was assigned.

In cases where the study auditor identified a potential discrepancy in interpretation between the radiology report and the radiographer PIE, the specific case was flagged for a second read by a senior consultant radiologist (with over 10 years experience). If a discrepancy was identified, the same (above) marking criteria would then be re‐applied to compare the PIE to the new, re‐read radiology report.

### Data analysis

Conventional descriptive statistics (number, percentage; median, interquartile range [IQR] and range) were used to describe participants’ demographic information. Resulting TP, TN, FN, FP and fractions (whole and partial) were summed, and sensitivity, specificity and accuracy percentages were calculated using the accepted formulae.^22^ This study reports two types of accuracy. One type relates to the level of accuracy of PIEs that were documented by the radiographers (referred to as ‘accuracy’). The other type relates to the accuracy of the entire PIE service (referred to as ‘service accuracy’). This was calculated by subtracting the percentage of cases where the radiographer had not provided a PIE (non‐participation) and the cases where the radiographer was unsure whether the examination was normal or abnormal, from the PIE accuracy. The overall ‘service accuracy’ was calculated as the authors believed it was more of an accurate representation of the PIE service efficacy holistically.

Prior to analyses, inter‐rater and intra‐rater reliability were calculated on a subset of the sample to ensure that the marking of examinations by the auditor was reliable. This involved an additional radiographer and the original auditor re‐marking 5% of the total sample (*n* = 315). Favourable inter‐rater reliability (kappa > 0.80 for all cases) and intra‐rater reliability (kappa > 0.90 for all cases) was observed, indicating a reliable marking process.

## Results

A total of 35 radiographers participated in the 12‐month study. The range of experience was from 1 to 35 years. 19 (54%) participants were female. The median (inter‐quartile range) years of radiographer experience was 7 (4–9.5).

**Figure 4:**
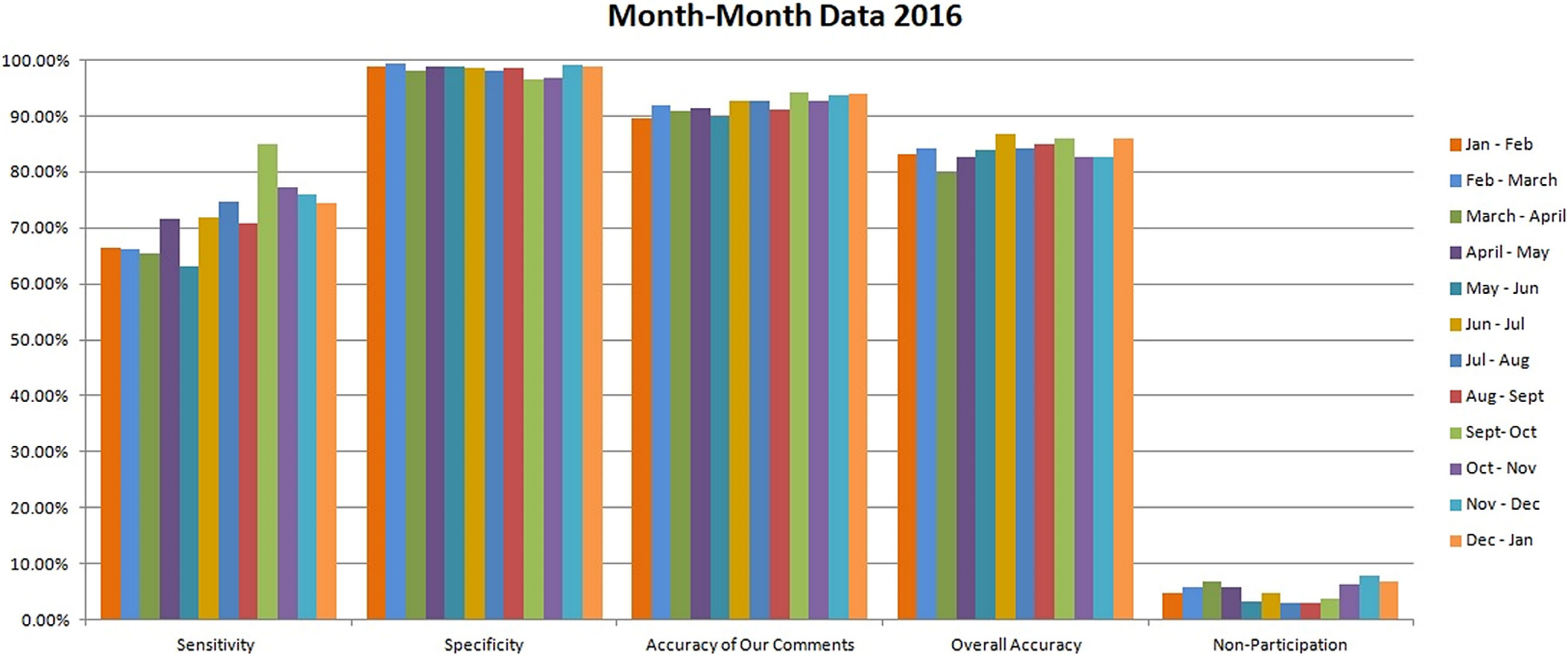
Month by month results.

Over the 12‐month study period 6290 examinations were reviewed. The mean ± 95% confidence level (standard deviation) for sensitivity, specificity and accuracy was 71.1% ± 2.4% (6.1), 98.4% ± 0.04% (0.9) and 92.0% ± 0.68% (1.9) respectively (Figure 4). The study reported an overall service accuracy of 84.1%. The mean (standard deviation) for non‐participation rate and unsure rate was 5.1% (1.6) and 3.6% (0.14) respectively.

Accuracy, false‐negative and false‐positive presentations are presented in Table 2. Given sensitivity (71.1%) was significantly lower than specificity (98.4%), false‐negative studies present the principle area for improvement and will be discussed in depth later. The five regions with the least false‐negative exams were skull, ribs, mandible, femur and calcaneus; all with 0. The five body parts with the most false‐negative exams were hand, foot, elbow, wrist and ankle with 50, 46, 42 and 42 respectively.

**Table 2:** Anatomical region breakdown

Anatomical region	False negative	False positive	Total	Accuracy, %
Abdomen	0	0	34	100.00
Ankle	40	10	524	90.33
Cervical spine	1	3	98	95.74
Calcaneus	0	0	16	100.00
Chest	19	4	914	96.91
Clavicle	3	0	27	88.89
Elbow	42	4	447	89.57
Facial bones	1	0	3	66.67
Femur	0	0	48	100.00
Fingers	21	0	203	89.55
Foot	46	7	472	88.40
Forearm	28	2	411	92.61
Hand	50	6	519	88.93
Hip	4	2	152	95.95
Humerus	5	0	60	91.67
Knee	19	2	416	94.68
Lumbar spine	10	1	158	92.31
Mandible	0	0	16	100.00
OPG	4	0	31	84.62
Pelvis	8	2	293	96.42
Ribs	0	0	8	100.00
Sacrum/Coccyx	5	0	10	50.00
Shoulder	34	11	432	89.31
Skull	0	0	1	100.00
Soft tissue neck	3	2	28	76.19
Sternum	2	0	18	88.89
Thoracic spine	3	0	82	96.05
Thumb	6	3	99	90.63
Tibia & Fibula	8	1	127	92.62
Toes	8	1	68	86.76
Wrist	42	4	335	86.10

Within the scope of this study, the anatomical region that recorded the most examinations of no PIE participation was the chest. It comprised 52.8% of all non‐participation items. Appendicular, axial and abdominal examinations equated to 30.3%, 16.3% and 0.6% of all non‐participation items respectively. The anatomical region with the most unsure items recorded was the elbow, with 11.5%.

Twenty‐one studies were flagged by the study auditor, where they believed an error in the radiologist's report was thought to have occurred. Of those studies, nine subsequent reviews revealed an abnormality that was described correctly by the radiographer and incorrectly by the original reporting radiologist. Any necessary patient follow‐up was led by the Radiologist through the ED treating team.

## Discussion

This is the largest study to report the accuracy of a radiographer PIE system in clinical practice. This investigation addressed its intended aim, by indicating that radiographers can provide a consistent PIE service that maintains a relatively high diagnostic accuracy. Such a service may compliment an emergency referrer's interpretation when a radiologist's report is unavailable. Thus, reducing the risk of patient harm in the emergency department due to undiagnosed plain imaging pathologies.

Given the overall objective of a radiographer PIE system is to support an emergency referrer in making accurate and timely treatment decisions, the authors believe that the service accuracy is the more valid representation of the service that is provided for the emergency referrer. At the end of the 12‐month study, PIE accuracy, when an interpretation was provided was 92.0%, whereas the overall PIE service accuracy was 84.1%. In clinical practice, the only available performance ‘benchmark’ is that of the radiologist. They are considered the international ‘gold standard’ in interpretation of radiographic images.^23^ The literature has acknowledged that radiologists have interpretative error rates between 3% and 6%, so a practical ‘benchmark’ for interpretation accuracy would be between 94% and 97%.^3^, ^24^, ^25^, ^26^ When comparing to the gold standard radiologist's report, it is apparent that the radiographer's PIE does not share the same accuracy. Albeit, a comparison to a radiologist's report is a substantially high benchmark, given radiographers have significantly less experience and education in radiographic image interpretation. Radiographers also review radiographic images on a lower resolution monitor and in an environment with brighter ambient lighting when compared with radiologists. Additionally, radiologists had access to the radiographer's PIE when reporting, however, it remains unclear whether it had any effect (positive or negative) on their reporting accuracy. Considering these differences, the results from this study highlight the potential for further improvement in radiographers’ image interpretation ability. This is paramount in order to effectively support the emergency referrer in providing optimal patient management. Furthermore, the service accuracy study period was consistent (range of 79.8–86.7%), which is encouraging (Figure 4). The small standard deviation for the mean sensitivity, specificity and overall accuracy provides further evidence of stability of the PIE service.

Interestingly, throughout the review of 6290 examinations, the nine examinations that were flagged for a review, revealed an abnormality that was described correctly by the radiographer and incorrectly by the original reporting radiologist. These reports were amended, and if required, any necessary patient follow‐up occurred. Interpretive discrepancies between two different professions, as experienced in this study, has been acknowledged in the literature.^8^ In McConnell et al.'s^8^ study, where they assessed the radiographic interpretation agreement between a group of radiographers and emergency doctors, on occasions both groups identified different pathologies for the same radiographic examination. Over the sample of examinations analysed in McConnell's study, when the combined accuracy of both professionals was calculated they were 1.2% higher than each group individually. This highlights that although the radiographer's PIE accuracy in this study was lower than the radiologist's, radiographers still provide valuable diagnostic information within their PIEs. Therefore, a PIE service has the ability to improve the emergency care service for patients. An interesting area for further research could involve investigating the types of discrepancies that exist between radiographers, radiologists and emergency referrers.

Although there is a paucity of published studies evaluating a radiographer PIE system in clinical practice, a number of studies have investigated PIE.^9^, ^10^, ^15^, ^27^, ^28^ This study's sensitivity (71.14%) was the lowest of the compared studies (85.55%, 74.40%, 95.00%, 93.50% and 91.80%) and its specificity (98.44%) was the highest of the compared studies (64.98%, 51.40%, 92.00%, 82.90% and 83.79%). When comparing this study's findings with the literature, several important methodological differences should be noted that could have influenced these findings. This study was undertaken during an operational PIE service within an emergency department. The study included all radiographers who worked in the emergency department and it analysed the entire 24‐hour period of selected days (comprised of day shift, evening shift and night shift). In comparison, several studies adopted a controlled, exam‐like evaluation of PIE accuracy in laboratory (non‐clinical) conditions.^9^, ^10^, ^15^, ^27^, ^28^ Additionally, in these five studies, all participating radiographers were handpicked based on experience and clinical expertise and the image assessment undertaken involved hand‐selected examinations to ensure a range of anatomical regions and pathologies were included. Two studies that investigated the effect of different methodological approaches of assessing radiographic image interpretation performance,^29^, ^30^ concluded that designing outcome measures under controlled conditions which contain image banks of a high abnormality prevalence may over‐estimate observer accuracy. This may provide evidence as to why the sensitivity calculated in this study was lower when compared with the controlled studies.^9^, ^10^, ^15^, ^27^, ^28^ The lower sensitivity and higher specificity rates of this study may demonstrate a more accurate reflection of radiographer ability than other studies. Further comparisons of differing methodological approaches to assessing radiographer PIEs as a viable and accurate complimentary image interpretation service would be of interest for further research.

The results of this study indicate several avenues that may improve service accuracy. The reported sensitivity (71.14%) and specificity (98.4%) indicates the radiographers demonstrated a high specificity when attempting to identify a normal study, but indicates room for improvement in examinations which contain an abnormality (sensitivity). Hand, foot, elbow, wrist and ankle examinations contained the most missed abnormalities (false negatives). This indicates that further education incorporating the use of search strategies and common sites of abnormalities (such as fractures) within the abovementioned anatomical regions may improve sensitivity. Overall, fractures of the phalanges (fingers and toes) are the most commonly missed pathologies. The authors recommend the analysis of pathology types (e.g. distal radius, phalangeal, calcaneus) as opposed to anatomical regions alone, this will provide a more accurate depiction of radiographer interpretation discrepancies. This is evidenced by phalangeal fractures having been reported in finger, hand, toe and foot radiographic series. A similar finding was noted for the next most commonly missed pathology, distal radius and ulna fractures. These fractures were reported in hand, wrist and forearm series. By targeting education in relation to these false negatives, hopefully an increase in the accuracy of the PIE system would occur. It is noteworthy that the anatomical regions that recorded the most missed abnormalities are also amongst the most commonly performed examinations in this study and outside this study.^31^

Although it would have been interesting to analyse the data further to identify any common patterns in patient demographics data was not collected on patient age or sex. This could be an area to explore in subsequent studies.

A further strategy to improve service accuracy would be to focus on reducing the unsure and non‐participation rates of radiographer PIEs. Analysis of these rates with respect to each anatomical region revealed chest examinations are responsible for 34.8% of all ‘unsure’ and ‘no PIE provided’ examinations. This was the largest contributor of any anatomical region. The authors’ opinion on why radiographers’ participation rates on interpreting chest examinations were significantly less than other regions is twofold. Firstly, confidence in interpreting chest radiographs is perceived to be low, due to the presence of potentially subtle, numerous and diverse pathologies that can manifest in a chest radiograph.^7^, ^32^ Secondly, and most important is the potential lack of understanding or awareness by radiographers of the pathologies that pertain to the chest that exist within the scope of this PIE system. Most commonly, when a chest radiograph was requested by an emergency referrer, several pathologies were documented to be excluded. Some of these pathologies were frequently within the scope of the PIE and some may have been outside. When radiographers overlooked pathologies that were within scope they selected the ‘outside scope’ option. This resulted in a non‐participation score being recorded by the auditor. Considering this finding, improvements can be made to further radiographers’ education and awareness of the pathologies that exist within the scope of this PIE system. This will potentially improve the overall service accuracy.

There are several notable strengths and limitations of this study. The sample size methodology employed may be considered a strength, given that the study reviews 14.7% of the total ED examinations performed annually. This exceeds a recommended audit sample size of 13.7%. Auditing only 2 days per week, although 1 weekday and one weekend day, could be considered a limitation given the same days each week were audited for consistency. Any variations to imaging activity which are specific to certain days may have gone unreported. To avoid this potential bias, the audit days could be alternated through different days of each week. Another limitation of the study was that individual accuracies of radiographers were not recorded. This restricts ascertaining whether certain radiographers may require more education than others. However, the shift roster at the study site was not cyclic and all participating radiographers worked a variety of shift times (morning, afternoon and nightshift). Therefore, it can be assumed that all participating radiographers contributed equally to the overall accuracy and did so by working a variety operational hours. Although radiologist feedback on comment quality and accuracy was received by the lead senior radiographer and disseminated to staff, no image interpretation education was conducted by the radiologists. All 17 image interpretation modules were created by the radiographers and approved by the lead senior radiographer in the emergency department before circulating to radiographers to complete. The lead senior radiographer had over 20 years of clinical experience. They also had completed a Masters degree in image interpretation and regularly lectured undergraduate university students on image interpretation. Not involving radiologists in the development of the education might be considered a limitation given the radiologist is known as the expert in interpreting medical imaging. Further and ongoing education annually, highlighting areas of low PIE accuracy would be prudent in improving the service's accuracy.

An interesting area for further research could investigate recording the management of patients where the radiologist's report was found to be incorrect. In particular, it would be interesting to identify whether the management was based on a decision that matched the radiographer PIE. Another area future research that warrants investigation would involve developing a more sustainable audit in comparison to the labour‐intensive audit described in this study. One approach could involve comparing the results of this study with a clinical audit that utilises a smaller sample size while maintaining similar robust methodology. Additionally, it would be interesting to collect data that records the time that the radiographer PIE was made. This would allow an analysis to be conducted to determine whether there is a specific time of the day when PIEs were more or less accurate. This would be of benefit to many clinical departments that are looking at implementing or monitoring a PIE system.

## Conclusion

This study has demonstrated that radiographers who were involved in this study provided a consistent PIE service while maintaining a reasonably high diagnostic accuracy. This form of image interpretation can complement an emergency referrer's diagnosis when a radiologist's report is unavailable at the time of patient treatment. PIE promotes a reliable enhancement of the radiographer's role with the multi‐disciplinary team. Ideally, this patient safety mechanism can and should be implemented within all emergency departments.
